# The Effect of Prescription Drugs and Alcohol Consumption on Intimate Partner Violence Victim Blaming

**DOI:** 10.3390/ijerph17134747

**Published:** 2020-07-01

**Authors:** Gemma Sáez, Manuel J. Ruiz, Gabriel Delclós-López, Francisca Expósito, Sergio Fernández-Artamendi

**Affiliations:** 1 Departamento de Psicología, Universidad Loyola Andalucía, 41704 Sevilla, Spain; gsaez@uloyola.es (G.S.); gdelcloslopez@al.uloyola.es (G.D.-L.); sfernandez@uloyola.es (S.F.-A.); 2 Department of Psychology and Anthropology, Education Faculty, University of Extremadura, 06006 Badajoz, Spain; 3 Mind, Brain and Behavior Research Center, University of Granada, 18011 Granada, Spain; fexposit@ugr.es

**Keywords:** alcohol, attitudes, intimate partner violence, prescription-drug, victim blaming

## Abstract

Intimate Partner Violence (IPV) is a public health problem with harsh consequences for women’s well-being. Social attitudes towards victims of IPV have a big impact on the perpetuation of this phenomenon. Moreover, specific problems such as the abuse of alcohol and drugs by IPV victims could have an effect on blame attributions towards them. The aim of this study was to evaluate whether the external perception (Study 1) and self-perception (Study 2) of blame were influenced by the victims’ use and abuse of alcohol or by the victims’ use of psychotropic prescription drugs. Results of the first study (N = 136 participants) showed a significantly higher blame attribution towards female victims with alcohol abuse compared to those without it. No significant differences were found on blame attributed to those with psychotropic prescription drugs abuse and the control group. Results of the second study (N = 195 female victims of interpersonal violence) showed that alcohol consumption is associated with higher self-blame and self-blame cognitions among IPV victims. However, results did not show significant differences on self-blame associated to the victims’ use of psychotropic prescription drugs. Our findings indicate that alcohol consumption, but not prescription drugs use, plays a relevant role in the attribution of blame by general population and self-blame by victims of IPV.

## 1. Introduction

Intimate partner violence (IPV) is defined as the violence committed by a current or former partner [1,2] and it includes any behavior “*that causes physical, sexual or psychological harm, including acts of physical aggression, sexual coercion, psychological abuse and controlling behaviors*” [3]. Worldwide, almost one third of women who have been in a romantic relationship report having experienced some form of physical and/or sexual victimization by their partner in their lifetime [4]. In fact, the 2030 Agenda of Sustainable Development Goals (SDGs) encourages all countries to eradicate IPV, since violence can negatively affect women’s physical, psychological and sexual wellbeing, increasing the risk of a poorer health, and it is associated with childhood trauma [5,6]. The most severe consequence of IPV is death, and 38% of the female murders are currently attributed to this form of violence [4]. Moreover, IPV can also have a negative impact on women’s economic and social outcomes [7], as well as on mental health (e.g., depression; [8]), which might eventually turn into alcohol and drugs abuse [9].

IPV victims blame themselves for the violence they have experienced [10]. Moreover, they are judged as more blameworthy by the society when they have violated gender roles [11]. Such feelings of blame have an important role on the perpetuation of this phenomenon, and they are related to adversely negative mental health consequences, including substance abuse [12]. However, the relation between IPV and substance use and abuse remains understudied. Data indicates that, among welfare recipients, victims of recent physical partner violence show higher rates of alcohol dependence (8%), compared to those who experienced physical partner violence in the past (2.6%) and those who have not experienced physical partner violence (1.1%) [13]. Women exposed to IPV have also shown to be more likely to use psychotropic drugs, even after controlling for mental health [14]. According to research, substance use might become a coping strategy for facing continuous violent experiences [15,16] since victims expect that substances will alleviate the negative physical and psychological impact of IPV [17]. Moreover, psychological violence has shown to undermine adaptative coping strategies such as social support among victims of IPV, leading to drug abuse problems [18]. Gezinki, Gonzalez-Pons and Rogers [9] concluded that survivors use alcohol and self-medication as a coping mechanism while abuse is occurring, in order to reduce pain; and after the relationship, victims use alcohol and self-medication as a way of coping with the trauma and the stress of building a new life. However, Kaysen and collaborators [19] have shown that the use of alcohol as a coping strategy may act as a mediator in the relation between alcohol use and trauma symptoms among IPV victims. In accordance with this approach, alcohol and drugs have been commonly understood as a consequence of the violence [17,20].

### 1.1. Social and Self-Perceptions of IPV Victims

One additional factor aggravating the negative consequences of IPV is social attitudes. As Flood and Pease [21] asserted: “attitudes play a role in the perpetration of this violence, in victims’ responses to victimization, and in community responses to violence against women (p. 125)”. These authors assert that women’s response to intimate personal violence is determined by their attitudes towards IPV as well as by the attitudes of those around them. Social attitudes towards intimate partner violence influence women’s perception of support and help in an IPV situation [22]. Stereotypic beliefs about intimate partner violence influence people’s evaluation of the victim, and “cultural beliefs around IPV de-legitimize individuals who have experienced partner abuse, often blaming those who have experienced IPV for their own victimization” [23] (p. 3).

More specifically, one critical attitude towards IPV victims in the general population is victim-blaming, which is defined as the process in which the victim is overtly or covertly attributed fault for their actions or inactions as the reason for the negative outcome [24]. Victim-blaming provokes that women not fitting into the traditional gender roles are negatively judged for the violence they are suffering [25]. Since the stereotypical features of an IPV victim are passivity and weakness [26], any deviation of such stereotypical view increases victim-blaming. Accordingly, empirical evidence has shown that women are judged more harshly in situations of sexual violence if they are behaving against traditional gender norms [21,27,28]. Viki and Abrams [29] demonstrated that female victims of sexual violence who have violated traditional gender roles are highly blamed by individuals who endorse sexism. Therefore, gender norms are limiting women’s acceptable behaviors and individual choices [11].

Victim-blaming is also important given its effect on external support provided in IPV situations. Victim-blaming has shown to be the mediator in the relationship between social attitudes and intervention in IPV situations [30]. When IPV victims are blamed for the violence experimented, they will encounter more obstacles when asking for help. Firstly, because they might self-blame themselves. Secondly, because they will anticipate that such social victim-blaming will turn into lower help. Moreover, female victims not fitting in the traditional gender roles are negatively judged in terms of blame [31]: “women who fail to conform to traditional expectations, either based on their occupations or their actions, are perceived as less warm and this lack of warmth leads to more blame and negativity in a psychological abuse context” [31] (p. 849).

Self-blame is defined as the attribution of the cause of a traumatic event to one’s own actions, assuming the responsibility of the event [32] and hindering recovery [32,33]. Self-blame is a criterion for Post-Traumatic Stress Disorder (PTSD) in the Diagnostic Statistic Manual Fifth version (DSM-5) [34] and plays an important role on intimate partner victimization (physical, psychological and sexual abuse). According to Andrews and Brewin [35], attribution of blame in individuals that have suffered traumatic violent experiences is a learned process where they may be more likely to blame their character, which is linked to lower levels of self-esteem. Reich and collaborators [32] have confirmed that self-blame has negative psychological consequences on IPV victims. In addition, according to the Transtheoretical Model of Behavior Change [36] internal blame attribution has been associated with the pre-contemplation phase in IPV victims. Moreover, women focused on feelings of guilt and self-blame have higher substance abuse and stay in the abusive relationship for a longer period of time [12]. As a consequence, internal attribution of blame enhances the mental health negative effects of IPV among victims, including higher levels of depression and lower levels of self-esteem, damaged psychological adjustment [12,32,37] and lower perceived social support.

### 1.2. Victim Blaming and Its Relation with Alcohol and Drug Use

Being male or female entails different expectations about social behaviors [38]. Gender has shown to be a crucial variable determining drug use as socially acceptable or unacceptable [39,40]. In this line, Brabete, Sánchez-López, Cuéllar-Floresa and Rivas-Diez [41] showed a negative relation between alcohol consumption and traditional female gender norms. Although a traditional feminine gender role might work as a protective factor for adverse health habits, it might also be detrimental for social perception and victim blame. In fact, previous studies have shown that women who use drugs report high levels of self-blame, shame, depressive mood, anxiety and tend to use substances more frequently alone and in private rather than in public settings [42]. Moreover, according to Capezza and Arriaga [31], non-traditional women are blamed more when they are attributed a lack of warmth. Following the Stereotype Content Model [43], drug users would be considered low in competence and low on warmth, labeled as completely cold [44]. This means that women using substances would face an increased stigma since they go against expected female roles.

In this direction, since a man drinking alcohol is more socially acceptable than a woman drinking alcohol [45], drinking among IPV victims will increase victim blame. Romero-Sánchez et al. [46] showed that the victim’s alcohol consumption influences victim blaming to a higher extent than the use of physical strength by the perpetrator. Moreover, victims who use substances are perceived as less truthful, leading to higher victim blaming [47]. Another study by Stewart and Maddren [48] showed that drunk victims of family violence were blamed more than non-drunk victims. However, despite previous studies suggesting the effect of drinking on IPV victim-blaming, no previous research has explored the differential effect of alcohol intake and other psychotropic drugs use on victim-blaming among IPV victims.

### 1.3. Overview of the Present Work

The current study aims to explore the effect of prescription drugs and alcohol use on intimate partner violence victim blaming. Our study has two different goals: (1) to assess the perception of the general population of women victims of IPV that use either alcohol or prescription drugs and (2) to determine whether there is a relationship between the use of alcohol/prescription drugs and self-blaming among IPV victims. With this design, we took a two-sided approach to examine how alcohol and prescription drugs influence intimate partner violence victim blaming.

Given that substantial research has shown the importance of social perception in the use of both legal and illegal drugs [49], the first study used an experimental design to explore the detrimental effect of alcohol abuse and prescription drugs abuse on the social perception of the IPV victim. 

Considering gender roles on drinking and drugs is of great importance [50], in the second study, we took a needed gender perspective because “the gender lens asks us to study substance use more carefully and to recognize the impact of social and cultural constructions of masculinity and femininity on individual and group drug use” [51] (p. 286). This gender perspective is necessary since alcohol use is significantly higher among men [50] whereas psychotropic drugs use is higher among women [52,53,54]. Therefore, a correlational study was carried out among victims of intimate partner violence to evaluate whether alcohol and psychotropic drug consumption are positively related to self-blaming. 

In order to understand the role of alcohol and psychotropic prescription drugs on IPV victim blaming from a social and victim perspective, two studies were carried out, with two different samples and two different recruitment strategies. The first experimental study was carried out with a paper-and-pencil technique, which allows for a better control of confounding variables and environmental distractors by the experimenter. The second study used an online sampling method for recruiting women meeting the established inclusion criteria. In this case, the online sampling method facilitated a higher perception of anonymity, which was crucial for the study given the sensitivity of the topic [55].

### 1.4. Hypotheses

The hypotheses of the first study are:

**Hypothesis** **1** **(H1).** *Victim-blaming would be higher when an alcohol abuse problem existed in comparison to a control condition without an alcohol abuse problem*.

**Hypothesis** **2** **(H2).** *Victim-blaming would be higher when the victim was an abuser of prescription drugs compared to a control condition without prescription drug abuse*.

The hypotheses of the second study are:

**Hypothesis** **3** **(H3).** *Victims of IPV that have an alcohol abuse problem would report higher levels of self-blaming (Hypothesis 3a) and higher self-blame cognitions (Hypothesis 3b) than those women without an alcohol abuse problem*.

**Hypothesis** **4** **(H4).** *Alcohol consumption is positively related to self-blaming (Hypothesis 4a) and self-blaming cognitions (Hypothesis 4b) among IPV victims*.

**Hypothesis** **5** **(H6).** *Victims of intimate partner violence who have ever used prescription drugs would self-blame themselves more (Hypothesis 5a) and would have higher self-blaming cognitions (Hypothesis 5b) compared to women who do not report any prescription drugs use*.

**Hypothesis** **6** **(H6).** *There is a positive correlation between prescription drugs use duration and self-blaming (Hypothesis 6a) and self-blaming cognitions (Hypothesis 6b) among IPV victims*.

## 2. Study 

### 2.1. Participants

A total of one hundred and fifty-five participants participated in the study. Nineteen of them were excluded from the analysis due to invalid data, leaving a total of 136 participants. The majority of participants self-identified as heterosexual (94.9%), and 52.2% were women. On average, participants were 30.40 years old (SD = 13.61). Modal education was a university degree (58.1%).

### 2.2. Procedure

The research study entitled “psychosocial factors related to the perception of intimate partner violence” was approved by the Ethical Committee of Loyola University previous data collection. Following an incidental sampling procedure in different crowded places (e.g., libraries) of a Spanish city, a researcher requested the participants’ collaboration answering a paper-and-pencil study. All participants were volunteers and provided informed written consent, and no monetary incentives were provided for participation. A between-subjects manipulation was used, where two identical IPV scenarios were created, except for the manipulation of the alcohol or prescription drug abuse problem reported by the victim of the interpersonal violence. After a random assignment of the manipulation, victim-blaming was assessed embedded within a larger series of questionnaires.

### 2.3. Manipulation

Interpersonal Violence scenarios: Three scenarios were created to incorporate the experimental manipulation from Nguyen et al. [56] second scenario. This scenario described a fictitious dating violence situation between Carlos and Ana, who were involved in a romantic relationship. The scenarios varied exclusively in the information regarding Ana’s substance abuse (psychotropic prescription drug abuse condition, alcohol abuse condition and control condition). The scenario provided to participants was as follows: “Carlos and Ana have been dating for about four years, both are students at the university (Alcohol condition: and Ana has alcohol abuse problems) (Psychotropic prescription drug condition: and Ana has psychotropic prescription drug abuse problems). One day, Ana went out with her friends and then came over to see Carlos. Carlos had been working hard on a project for one of his classes. He had wanted Ana to help him with this task and was very angry at Ana her for going out with her friends. He was quite upset and slapped her fairly hard. She fell and broke a bone in her hand. This required a visit to the emergency room to reset the hand and to get pain pills” (adapted from Nguyen et al.; scenario 2 [56]).

### 2.4. Measures

Victim Blame: Six questions from Romero-Sánchez, Megías and Krahé [57] were used to assess victim blame: “Do you believe Ana should feel guilty for what happened at the end of this story?” “Do you believe Ana incited Carlos to act like he did at the end of this story?” “Do you believe Ana could have behaved differently to change the outcome of this story?” “Do you believe Ana got what she deserved?” “Do you believe Ana could have prevented what happened at the end of this story?” and “Do you think that Ana’s behavior provoked what happened at the end of this story?” The scale response format was a 7-point Likert-scale form, with responses ranging from (1) strongly disagree to (7) strongly agree. Higher scores indicated more blame attributed to the victim. There was strong internal consistency in the present study (Cronbach’s α = 0.81).

### 2.5. Data Analysis

All analyses were conducted using SPSS (Statistical Package for the Social Sciences Version 26.0, IBM Corp., Armonk, NY, USA) software. To evaluate the effect that experimental manipulation (control, prescription drug and alcohol) has on victim blaming, one-way ANOVAs with Welch’s test procedure was conducted. Additionally, pairwise comparison tests were used to examine descriptive data.

### 2.6. Results

Regarding the ANOVA for victim blaming with the different conditions (control, psychotropic prescription drug and alcohol), Levene’s test for heterogeneity of variance indicated that responses to victim blame questionnaire violated the homogeneity of variance assumption (F (2, 133) = 10.59, *p* < 0.001). Accordingly, we conducted Welch’s test procedure [58]. Welch’s test held a significant effect *F_Welch_* (2, 80.63) = 5.62, *p* = 0.005 (see Table 1). Our results confirmed Hypothesis 1, and pairwise comparisons indicated that participants who read the alcohol abuse scenario scored higher on victim-blaming compared to the control condition (*t* (59.97) = −3.19, *p* = 0.002). However, results did not confirm Hypothesis 2, and there were no significant differences on victim-blaming among participants who read the psychotropic prescription drug abuse scenario and the control scenario (*t* (78.56) = −1.78, *p* = 0.08). Means are presented in Table 1. Results did not yield a significant effect of the psychotropic prescription drug on the blame attribution to the victim. There was, however, a clear tendency among participants to attribute a higher blame to women who have a prescription drug problem compared to the control condition.

Victims of intimate partner violence are targets of a double source of blaming, namely: the social judgement about their victim status and their own perception of their responsibility of such violence. Results from the first study showed the importance of victim’s alcohol consumption on victim blaming among general population; the second study aims to explore the effect that alcohol and prescription drug use have on the victim’s self-blaming and self-blaming cognitions.

## 3. Study 2

### 3.1. Participants and Procedure

The study entitled “Alcohol and prescription drug consumption and its effect on interpersonal relationships” was approved by the Institutional Ethical Committee of Loyola University previous data collection and participants were recruited from an online survey. Inclusion criteria were: (1) self-identifying as a woman, (2) being older than 18, (3) currently in a heterosexual relationship, (4) correctly answering two attentional checks and (5) self-identifying as victims of IPV according to the Woman Abuse Screening Tool (WAST-Short; see measures section). Participants not meeting eligibility criteria were excluded from the analysis, resulting in a final sample of 195 female participants. On average, participants were 32.36 years old (SD = 14.33). Modal education was a university degree or Upper-Level Training Cycles (88.3% of participants).

### 3.2. Measures

The Woman Abuse Screening Tool (WAST-Short): This tool was utilized to evaluate the inclusion criteria of self-identifying as victims of IPV. The Spanish-language version of this scale has shown good specificity (76.2%) and good sensitivity (91.4%) [59]. The WAST-Short is composed of two items that assess the degree of relationship tension and the difficulty that the couple reports in solving arguments on a scale from 1 (no tension/no difficulty) to 3 (a lot of tension/great difficulty) [60]. Following Plazaola-Castaño et al. [59] a response of 2 or 3 on any of the two items was coded as positive (1) whereas a response of 1 was coded as negative (0). The total score, calculated by summing both items, ranges from 0 to 2, with 2 being the cutoff (participants scoring 0 or 1 were excluded). Consequently, participants in the study included those experiencing some or great difficulty and some or a lot of tension on their relationship. This way of calculating the final score is the recommended for Spanish population [59].

The Alcohol Use Disorders Identification Test (AUDIT) [61] is a 10-item self-report screening measure assessing Alcohol Use Disorders (AUD) and harmful alcohol use. The current study utilized the total score to measure alcohol use problems, with a cut-off of 7 points for problem drinking as suggested by Babor and collaborators [62] to be used by women. There was strong internal consistency in the present study (Cronbach’s α = 0.81).

Prescription drugs: In the absence of an existing validated measure of the extent to which participants use prescription drugs, we adapted four items from a larger measure used by Antich and collaborators [63]. Specifically, we asked participants: “Have you ever taken psychotropic prescription drugs for depression, anxiety or sleep disorders?” with a response format including 0 (no consumption) or 1 (consumption). Two participants have missing data in this question. Among participants reporting any psychotropic drug use, three different questions were presented to explore the length of time that they had been using prescription drugs for (a) depression, (b) anxiety and (c) sleep disorders. With this purpose, a Likert scale was used with responses including 1 (less than 1 month), 2 (from 1 to 3 months), 3 (more than six months) and 4 (more than one year). The final timeframe of prescription drug consumption was the average of the time reported for depression, anxiety and sleep disorders (M = 2.43; SD = 1.04). This result indicates that among participants who reported any prescription drug use, they had done so for between three and six months in average. A total of eight responses were coded as missing on these two measures because of the inconsistent responses of participants to the items (e.g., reporting no previous psychotropic prescription drug use in the first question but then providing details on the specific features of their prescription drug use).

Self-blame: In order to evaluate the self-blame that women feel as a consequence of being victims of interpersonal violence, the combination of two instruments was used. Firstly, the items of the psychological violence (e.g., my partner demands obedience to his whims) from the Reduced Spanish version of the Index of Spouse Abuse were used (ISA) [64], and afterwards, the level of self-blame that female participants felt towards these items was assessed. Secondly, we listed the items referring to behaviors of physical violence (e.g., my partner becomes abusive when he drinks) from the ISA scale and immediately asked participants about the level of self-blame regarding those items. Specifically, after reading the items on physical or non-physical violence, we asked participants: “How guilty do you feel?”. The response format is a 6-point Likert ranging from 0 (not guilty) to 5 (totally guilty). We calculated the total score by averaging the score reported for both items. There was strong internal consistency of this measure in the present study (Cronbach’s α = 0.85).

Self-blame cognitions: The subscale from the posttraumatic cognitions Inventory (PTCI) [65] was translated (and back-translated) into Spanish and adapted for the study purpose. Self-blame subscale is a self-reported measure to assess self-blame as part of the traumatic cognition, and it was adapted to evaluate female perception of having acted wrongly with regards to the violence they have experienced (e.g., “It happened because of the way I acted”; “It happened to me because of the sort of person I am”). The response format is a 7-point Likert from 1 (totally disagree) to 7 (totally agree). The total score was calculated as the mean of the five items [66]. There was strong internal consistency in the present study (Cronbach’s α = 0.87). The self-blame questions of the trauma questionnaire were presented immediately after the items from the Reduced Spanish version of the Index of Spouse Abuse [64]. The self-blame cognition subscale was adapted in order to ask participants to answer these questions with regards to the victimization items they had just read.

### 3.3. Data Analysis

Independent samples t-tests were conducted for testing Hypothesis 3, predicting significant differences on self-blame (Hypothesis 3a) and self-blame cognitions (Hypothesis 3b) depending on their alcohol problematic consumption. To test Hypothesis 4, which predicts that alcohol consumption will predict self-blame (Hypothesis 4a) and self-blame cognition (Hypothesis 4b) among IPV victims, regression analyses were run to evaluate the predicting role of self-blame and self-blame cognitions on alcohol use. To test Hypothesis 5, which predicts differences on self-blame (Hypothesis 5a) and self-blame cognition (Hypothesis 5b) among IPV victims depending on their prescription drug use, independent samples t-tests were conducted. Lastly, in order to test Hypothesis 6, predicting a relation between prescription drugs use duration and self-blaming (Hypothesis 6a) and self-blaming cognitions (Hypothesis 6b), correlation analysis between drug use duration and both measures of self-blaming were conducted.

### 3.4. Results

Descriptive statistics and correlations are reported in Table 2 and Table 3. Hypothesis 3, which predicts higher self-blame (Hypothesis 3a) and self-blame cognition (Hypothesis 3b) on IPV victims with an alcohol abuse problem, is partially supported (see Table 3). In accordance with Hypothesis 3a, female participants who reported having problematic alcohol consumption scored higher on self-blame (*t* (43.26) = −2.22, *p* = 0.03). Women scoring above the alcohol problematic cutoff scored higher on self-blame (M = 0.81, SD = 1.31) compared to women below the cutoff (M = 0.31, SD = 0.81). However, and contrary to Hypothesis 3b, female participants who reported having a problematic alcohol consumption did not report higher self-blaming cognitions (*t* (177) = −0.99, *p* = 0.33) (see Table 4).

Our results confirmed Hypotheses 4a and 4b, since higher alcohol consumption was related to higher self-blame (*b* = 0.22, *t* = 2.97, *p* = 0.003) and self-blame cognitions (*b* = 0.17, *t* = 2.23 *p* = 0.03) (see Table 5).

Finally, our results did not allow us to confirm Hypotheses 5a and 5b (see Table 4). There were no significant differences between female participants on their self-blame (*t* (175) = 0.09, *p* = 0.93) and their self-blame cognitions (*t* (173) = −1.53, *p* = 0.13) depending of their prescription drug consumption. Moreover and contrary to Hypothesis 6, which predicts a positive relation between the prescription drug duration and self-blame (6a) and self-blame cognition (6b) among IPV victims, there was not a significant association between time consumption of prescription drugs and self-blame (*r* = 0.13, *p* = 0.27) and self-blame cognitions (*r* = −0.04, *p* = 0.71) reported by victims of intimate partner violence.

## 4. Discussion

The present work examined the effect of alcohol and prescription drug use on victim-blaming. Particularly, we have focused on female victimization since the World Health Organization Report [3] states that women are frequent targets of violence, whereas men are more likely to be perpetrators. Moreover, social perception in intimate partner violence phenomena is determinant [67]. With these considerations in mind, the present study had two goals in different populations. Firstly, we examined the social perception of victim-blaming depending on the victim’s alcohol or prescription drug abuse. Secondly, and focusing on intimate partner violence victims, we evaluated whether the reported consumption of alcohol and prescription drugs would be related to self-blame and self-blaming cognitions.

Study 1 revealed that presenting an alcohol abuse problem but not a prescription-drug abuse problem was related to higher victim-blaming compared to a control condition among general population. This result might be explained because women who use alcohol are considered to violate social gender norms, whereas prescription drug abuse is in accordance with the stereotypical view of women as emotionally weak [26]. This result replicates previous studies reporting that *“drinking by domestic violence victims would lead to increased victim blame and derogation”* [45] (p. 1054) because alcohol consumption has a detrimental effect on the attribution of empathy and compassion. Alcohol abuse causes women to be perceived as behaving inappropriately, which decreases the perpetrator’s attribution of blame and increases the female’s responsibility of the violence [68,69]. From the perspective of the Just World Theory [70], which states that bad things happen just to bad people and people who deserve it, victim blaming in intimate partner violence situation is one of the strategies that allow people to believe that the world is a just and fair place [71]. Valor-Segura, Expósito and Moya [72] found that people who endorse to a higher extent the Belief in a Just World attribute higher victim-blaming to the victim when there is not a cause for the violence, because people tend to think that the victim must have done something to deserve such violence. Our experimental manipulation gave participants a pretext to attribute blame to the victim, i.e., she has an alcohol problem.

Moreover, the current work extends previous literature revealing that there are no differences on victim blame associated to the victim’s psychotropic prescription drugs abuse problem. This result might be explained because the stereotype of women abusing prescription drugs would be that of weakness and overemotional among other characteristics [73], which is in accordance with the submissive stereotypical position hold by intimate partner violence victims. This result is in line with findings from Little and Terrance [74], supporting that female victims described as more feminine are considered less blameworthy. Merging results from Capezza and Arriaga [31] and the findings presented here, we can assert that women with an alcohol abuse problem are violating their gender role, and turning into higher victim-blaming in case they experience IPV violence.

Does social stigma towards female victims of IPV with alcohol abuse problem affect their own perception and attitudes toward the violence they experience? Based on the fact that women with substance abuse problems perceive lower levels of social support in the context of IPV experiences [75], we hypothesized that among victims of IPV with higher drugs and alcohol consumption, the internalization of social blaming would be higher compared to IPV victims with lower substance use. With this goal in mind, the second study using a specific sample of IPV victims aimed to explore the effect that alcohol and prescription drugs have on self-blaming attribution for the violence they had experienced. Results of Study 2 concluded that victims with higher alcohol consumption or an alcohol use disorder endorse submissive and self-blaming beliefs. This result is in line with previous studies indicating that victims of intimate partner violence with addiction problems have a lack of personal autonomy [76].

This result is highly relevant because alcohol abuse is significantly higher among those women who have experienced intimate violence compared to non-victims [77]. If experiencing violence is a risk factor for alcohol abusive problem, and this abusive behavior leads to self-blaming among IPV victims, self-blaming is a perpetuating phenomenon among women who use alcohol as a coping strategy for IPV situations. According to the trans-theoretical model, self-blaming cognitions and feelings might contribute to the victims’ emotional distress and hinder victims’ progression through the TTM-based stages. The use of alcohol by IPV victims might aggravate this situation. Moreover, it might be that alcohol consumption is used by female victims as a way of enhancing the bond with their partner [78], given the high emotional dependence on their partners [79]. Another alternative is that their perception of lack of mate alternatives because of their addiction would decrease their readiness to leave the abusive relationship [78,80]. This might in turn increase their self-blame for not leaving the abusive relationship. Lastly, another alternative is that self-blaming cognitions might be explained because women are aware that they are violating the gender norm due to their alcohol consumption. This perception comes from social stigmatization to female drinkers [81]. However, they would not be aware of such violation if they use psychotropic prescription drugs since its consumption is perceived as normalized and harmless, because they might be prescribed by a doctor [82].

## 5. Limitations

Despite the significant findings in our study about the role of alcohol and prescription drugs use on blame and self-blaming attribution among IPV victims, this study is not without limitations. Firstly, the current studies focused solely on one side of the phenomenon. While gender violence is a gendered phenomenon where women are mostly victims and men are perpetrators of the violence, it would be important to evaluate the level of male alcohol consumption to control for relations found between self-blaming and alcohol consumption in Study 2.

Secondly, the manipulation in the first study has been carried out through a scenario method, which might have limitations regarding external validity. Additionally, it was carried out with a convenience sample, and the level of victimization or perpetration of participants was not evaluated. Even though this level of victimization should be randomly distributed across the three conditions, future studies should explore the role of experiencing violence on victim blame attribution. Thirdly, the correlational nature of Study 2 avoids any interpretation of causality. Accordingly, longitudinal studies should be conducted that examine the long-term and reciprocal associations between IPV victim blaming on alcohol and prescription drugs use. Fourthly, the use of different samples for each study compels us to be cautious regarding the interpretation of possible relationships between studies. Further studies should evaluate both the effect of contextual and internal variables in the same sample of IPV victims and their social network. Fifthly, previous research indicates that variables such as depressive symptoms [83] or dysphoric symptoms [84] might also have a significant impact on self-blame and self-blaming cognitions. In our study, and due to time limitations, no further information was collected on possible control variables. Results from OLS regression should then be interpreted with caution.

Finally, an important limitation of the second study is the use of the WAST. In our study, we have used the criterion scoring for Spanish population to create the cut-off recommended by Plazaola-Castaño et al. [59] due to its higher sensitivity. We must note though that this cut-off level has been shown to detect a high number of false positives, meaning that some of the women who are considered as battered by the instrument might be erroneously classified as such. However, in our study, we are not particularly interested in an exhaustive detection of intimate partner violence, and our interest is to analyze the effect of alcohol and prescription drug use on self-perception among women experiencing any kind of violence.

## 6. Conclusions

This investigation is designed as a potential contributor to the attainment of the fifth goal of the Sustainable Development Goals (SDG) adopted by the United Nations Member States, which aims to achieve gender equality and empower all women and girls. Specifically, we have adopted a dual approach that integrates social and individual perspectives on the victim’s blame attribution analysis. This allows us to attain a comprehensive understanding of the role of alcohol and drugs on victim blaming that contributes to expand our knowledge on the role of victim’s behaviors on attributions. In particular, results revealed that alcohol consumption, but not prescription drug consumption, increases blame attribution among observers and self-blame attribution among victims. Both results are closely related because of the fact that people perceive women with an alcohol abuse problem as responsible for the violence they experience, it is manifested in everyday discriminant and non-helping behavior [85,86] causing the victim to internalize such blame.

Present findings and future studies in this line of research have implications for interventions with victims who are alcohol users and for policy intervention. On the one hand, the consideration of blaming attribution and self-attribution is crucial for mental health professionals working with IPV victims, since self-blame moderates the relation between physical abuse and PTSD severity [36]. Drinking problems on IPV victims need to be understood as a way of coping with self-blaming cognitions. On the other hand, policymakers working on awareness campaigns might find the results from study 1 useful for creating preventive programs aiming to reduce IPV by modifying individual attitudes towards victims with alcohol abuse problems. Lastly, since the current COVID-19 pandemic situation has shown to exacerbate IPV victimization [87], as well alcohol and drug problematic consumption [88,89], we encourage researchers to explore the effect that COVID-19 pandemic might have on the use of alcohol as a way of coping with IPV situations, due to the lack of social support.

## Figures and Tables

**Table 1:** Descriptive statistics and ANOVAs for victim blaming.

Variable	*n*	M(SD)	df	F*_Welch_*	*p*
Victim Blaming			2, 80.63	5.62	0.005
Alcohol condition	45	1.99 (1.29)			
Prescription drug condition	44	1.58 (0.78)			
Control condition	47	1.32 (0.57)			

Note: Welch’s statistics are reported because of violation of homogeneity of variance assumption.

**Table 2:** Descriptive statistics.

Variable	Range	Mean	SD	N
1. Self-blame	0–5	0.42	0.96	186
2. Self-blame cognitions	1–7	2.16	1.42	184
3. Alcohol consumption	0–25	4.39	4.43	189
4. Prescription drug consumption duration	1–4	2.43	1.04	78

Note: The different sample size is due to missing data in some of the scales.

**Table 3:** Correlations of Study 2.

Variable	1	2	3	4
1. Self-blame				
2. Self-blame cognitions	0.35 ***			
3. Alcohol consumption	0.22 **	0.17 *	--	
4. Prescription drug consumption duration	0.13	−0.04	−0.02	--

Note: * Correlation is significant at the 0.05 level (2-tailed); ** Correlation is significant at the 0.01 level (2-tailed); *** Correlation is significant at the 0.001 level (2-tailed).

**Table 4:** Descriptive statistics and paired *t*-test for alcohol consumption and prescription drugs of Study 2.

**Alcohol**						
	Below the Alcohol Cutoff		Above the Alcohol Cutoff			
Variable	*n*	M (SD)	*n*	M (SD)	*t (gl)*	*p*
Self-blame	143	0.31 (0.81)	37	0.81 (1.31)	−2.22 (43.26)	0.032 *
Self-blame cognitions	143	2.11 (1.38)	36	2.38 (1.65)	−0.99 (177)	0.326
**Prescription Drugs**						
	No prescription Drugs		Prescription Drugs			
Variable	*n*	M (SD)	*n*	M (SD)	*t (gl)*	*p*
Self-blame	101	0.44 (1.04)	76	0.43 (0.89)	0.09 (175)	0.93
Self-blame cognitions	99	2.02 (1.33)	76	2.35 (1.53)	−1.53 (173)	0.13

Note: * *p* < 0.05, significant difference; The difference in sample size is due to missing data on some scales.

**Table 5:** Regression Analysis of Study 2.

**Variable**	**Self-Blame**				
Predictor	B	*SE*	*t*	*p*	*n*
Alcohol consumption	0.05	0.02	2.97	0.003	179
Model Statistics	*df* (1, 178)	*F* 8.82	*R* 0.22	*R*^2^ 0.05	
	**Self-Blame Cognitions**				
Predictor	B	*SE*	*t*	*p*	*n*
Alcohol consumption	0.05	0.02	2.23	0.027	
Model Statistics	*df* (1, 177)	*F* 4.96	*R* 0.17	*R*^2^ 0.03	178

## References

[1] Breiding M.J., Basile K.C., Smith S.G., Black M.C., Mahendra R. (2015). Intimate Partner Violence Surveillance Uniform Definitions and Recommended Data Elements Version 2.0.

[2] (2003). National Center for Injury Prevention and Control Costs of Intimate Partner Violence against Women in the United States.

[3] World Health Organization (2010). Preventing Intimate Partner and Sexual Violence against Women: Taking Action and Generating Evidence.

[4] García-Moreno C., Pallitto C., Devries K., Stöckl H., Watts C., Abrahams N. (2013). Global and Regional Estimates of Violence against Women: Prevalence and Health Effects of Intimate Partner Violence and Non-Partner Sexual Violence.

[5] Loxton D., Dolja-Gore X., Anderson A.E., Townsend N. (2017). Intimate partner violence adversely impacts health over 16 years and across generations: A longitudinal cohort study. PLoS ONE.

[6] Fulu E., Miedema S., Roselli T., McCook S., Chan K.L., Haardörfer R., Jewkes R. (2017). UN Multi-country Study on Men and Violence study team Pathways between childhood trauma, intimate partner violence, and harsh parenting: Findings from the UN Multi-country Study on Men and Violence in Asia and the Pacific. Lancet Glob. Health.

[7] Campbell J.C. (2002). Health consequences of intimate partner violence. Lancet.

[8] Devries K.M., Mak J.Y., Bacchus L.J., Child J.C., Falder G., Petzold M., Astbury J., Watts C.H. (2013). Intimate Partner Violence and incident depressive symptoms and suicide attempts: A Systematic Review of longitudinal studies. PLoS Med..

[9] Gezinski L.B., Gonzalez-Pons K.M., Rogers M.M. (2019). Substance use as a coping mechanism for survivors of Intimate Partner Violence: Implications for safety and service accessibility. Violence Women.

[10] Scheffer Lindgren M., Renck B. (2008). “It is still so deep-seated, the fear”: Psychological stress reactions as consequences of intimate partner violence. J. Psychiatr. Ment. Health Nurs..

[11] Parent M.C., Moradi B. (2010). Confirmatory Factor Analysis of the Conformity to Feminine Norms Inventory and Development of an Abbreviated Version: The Cfni-45. Psychol. Women Q..

[12] Karakurt G., Smith D., Whiting J. (2014). Impact of Intimate Partner Violence on women’s mental health. J. Fam. Violence.

[13] Tolman R.M., Rosen D. (2001). Domestic violence in the lives of women receiving welfare: Mental Health, Substance Dependence, and Economic Well-Being. Violence Women.

[14] Kilpatrick D.G., Acierno R., Resnick H.S., Saunders B.E., Best C.L. (1997). A 2-year longitudinal analysis of the relationships between violent assault and substance use in women. J. Consult. Clin. Psychol..

[15] Clark H.W., Masson C.L., Delucchi K.L., Hall S.M., Sees K.L. (2001). Violent traumatic events and drug abuse severity. J. Subst. Abus. Treat..

[16] Simonelli A., Pasquali C.E., De Palo F. (2014). Intimate partner violence and drug-addicted women: From explicative models to gender-oriented treatments. Eur. J. Psychotraumatol..

[17] Martino S.C., Collins R.L., Ellickson P.L. (2005). Cross-lagged relationships between substance use and intimate partner violence among a sample of young adult women. J. Stud. Alcohol..

[18] Schultz K., Walls M., Grana S.J. (2019). Intimate Partner Violence and Health: The roles of social support and communal mastery in five American Indian communities. J. Interpers. Violence.

[19] Kaysen D., Dillworth T.M., Simpson T., Waldrop A., Larimer M.E., Resick P.A. (2007). Domestic violence and alcohol use: Trauma-related symptoms and motives for drinking. Addict. Behav..

[20] El-Bassel N., Gilbert L., Wu E., Go H., Hill J. (2005). Relationship between drug abuse and Intimate Partner Violence: A longitudinal study among women receiving methadone. Am. J. Public Health.

[21] Flood M., Pease B. (2009). Factors Influencing Attitudes to Violence against Women. Trauma Violence Abus..

[22] McDonnell K.A., Burke J.G., Gielen A.C., O’Campo P., Weidl M. (2011). Women’s Perceptions of Their Community’s Social Norms Towards Assisting Women Who Have Experienced Intimate Partner Violence. J. Urban Health.

[23] Overstreet N.M., Quinn D.M. (2013). The Intimate Partner Violence stigmatization model and barriers to help seeking. Basic Appl. Soc. Psychol..

[24] Harber K.D., Podolski P., Williams C.H. (2015). Emotional disclosure and victim blaming. Emotion.

[25] Gustafson A.L. (2005). Seminarians’ Response to Domestic Violence: Sex-Role Attitudes, Just World Beliefs, and Formal Training. Ph.D. Thesis.

[26] Dunn J.L. (2005). “Victims” and “Survivors”: Emerging vocabularies of motive for “Battered Women Who Stay”. Sociol. Inq..

[27] Golden J.H., Johnson C.A., Lopez R.A. (2001). Sexual Harassment in the workplace: Exploring the effects of attractiveness on perception of harassment. Sex Roles.

[28] Koepke S., Eyssel F., Bohner G. (2014). “She Deserved It”: Effects of sexism norms, type of violence, and victim’s pre-assault behavior on blame attributions toward female victims and approval of the aggressor’s behavior. Violence Women.

[29] Viki G.T., Abrams D. (2002). But she was unfaithful: Benevolent sexism and reactions to rape victims who violate traditional gender role expectations. Sex Roles.

[30] West A., Wandrei M.L. (2002). Intimate Partner Violence: A model for predicting interventions by informal helpers. J. Interpers. Violence.

[31] Capezza N.M., Arriaga X.B. (2008). Why do people blame victims of abuse? The role of stereotypes of women on perceptions of blame. Sex Roles.

[32] Reich C.M., Jones J.M., Woodward M.J., Blackwell N., Lindsey L.D., Beck J.G. (2014). Does self-blame moderate psychological adjustment following intimate partner violence?. J. Interpers. Violence.

[33] Moor A., Farchi M. (2011). Is rape-related self blame distinct from other post traumatic attributions of blame? A comparison of severity and implications for treatment. Women Ther..

[34] American Psychiatric Association (2013). Diagnostic and Statistical Manual of Mental Disorders.

[35] Andrews B., Brewin C.R. (1990). Attributions of blame for marital violence: A study of antecedents and consequences. J. Marriage Fam..

[36] Prochaska J.O., DiClemente C.C. (1982). Transtheoretical therapy: Toward a more integrative model of change. Psychol. Psychother..

[37] O’Neill M.L., Kerig P.K. (2000). Attributions of self-blame and perceived control as moderators of adjustment in battered women. J. Interpers. Violence.

[38] Eagly A.H., Wood W. (2012). Social Role Theory. Handbook of Theories of Social Psychology: Volume 2.

[39] Becker J.B., McClellan M., Reed B.G. (2016). Sociocultural context for sex differences in addiction. Addict. Biol..

[40] Courtwright D.T. (2001). Forces of Habit: Drugs and the Making of the Modern World.

[41] Brabete A.C., del Sánchez-López M.P., Cuéllar-Flores I., Rivas-Diez R. (2013). The impact of gender norms on alcohol and tobacco use at Romanians. Procedia Soc. Behav. Sci..

[42] Nelson-Zlupko L., Kauffman E., Dore M.M. (1995). Gender Differences in Drug Addiction and Treatment: Implications for Social Work Intervention with Substance—Abusing Women. Soc. Work.

[43] Fiske S.T., Cuddy A.J.C., Glick P., Xu J. (2002). A model of (often mixed) stereotype content: Competence and warmth respectively follow from perceived status and competition. J. Personal. Soc. Psychol..

[44] Cuddy A.J.C., Fiske S.T., Glick P. (2008). Warmth and competence as universal dimensions of social perception: The stereotype content model and the BIAS Map. Advances in Experimental Social Psychology.

[45] Harrison L.A., Esqueda C.W. (2000). Effects of race and victim drinking on domestic violence attributions. Sex Roles.

[46] Romero-Sánchez M., Megías J.L., Krahé B. (2011). The role of alcohol and victim sexual interest in Spanish students’ perceptions of sexual assault. J. Interpers. Violence.

[47] Abbey A., Zawacki T., Buck P.O., Clinton A.M., McAuslan P. (2004). Sexual assault and alcohol consumption: What do we know about their relationship and what types of research are still needed?. Aggress. Violent Behav..

[48] Stewart A., Maddren K. (1997). Police officers’ judgements of blame in family violence: The impact of gender and alcohol. Sex Roles.

[49] Lee N., Boeri M. (2017). Managing Stigma: Women Drug Users and Recovery Services. Fusio.

[50] Room R. (1996). Gender roles and interactions in drinking and drug use. J. Subst. Abus. Treat..

[51] Anderson T.L. (2003). Drug Use and Gender. Encyclopedia of Criminology and Deviant Behavior: Self-Destructive Behavior and Disvalued Identity.

[52] Boyd A., Van de Velde S., Pivette M., Ten Have M., Florescu S., O’Neill S., Caldas-de-Almeida J.-M., Vilagut G., Haro J.M., Alonso J. (2015). Gender differences in psychotropic use across Europe: Results from a large cross-sectional, population based study. Eur. Psychiatry.

[53] van der Heyden J.H.A., Gisle L., Hesse E., Demarest S., Drieskens S., Tafforeau J. (2009). Gender differences in the use of anxiolytics and antidepressants: A population-based study. Pharmacoepidemiol. Drug Saf..

[54] Kantor E.D., Rehm C.D., Haas J.S., Chan A.T., Giovannucci E.L. (2015). Trends in Prescription Drug Use Among Adults in the United States From 1999–2012. JAMA.

[55] Ward P., Clark T., Zabriskie R., Morris T. (2014). Paper/Pencil Versus Online Data Collection. J. Leis. Res..

[56] Nguyen T.T., Morinaga Y., Frieze I.H., Cheng J., Li M., Doi A., Hirai T., Joo E., Li C. (2013). College students’ perceptions of Intimate Partner Violence: A comparative study of Japan, China, and the United States. Int. J. Confl. Violence.

[57] Romero-Sánchez M., Krahé B., Moya M., Megías J.L. (2017). Alcohol-related victim behavior and rape myth acceptance as predictors of victim blame in sexual assault cases. Violence Women.

[58] Tomarken A.J., Serlin R.C. (1986). Comparison of ANOVA alternatives under variance heterogeneity and specific noncentrality structures. Psychol. Bull..

[59] Plazaola-Castaño J., Ruiz-Pérez I., Hernández-Torres E. (2008). Validación de la versión corta del Woman Abuse Screening Tool para su uso en atención primaria en España. Gac. Sanit..

[60] Brown J.B., Lent B., Schmidt G., Sas G. (2000). Application of the Woman Abuse Screening Tool (WAST) and WAST-short in the family practice setting. J. Fam. Pract..

[61] Saunders J.B., Aasland O.G., Babor T.F., de la Fuente J.R., Grant M. (1993). Development of the Alcohol Use Disorders Identification Test (AUDIT): WHO collaborative project on early detection of persons with harmful alcohol consumption-II. Addiction.

[62] Babor T.F., Higgins-Biddle J.C., Saunders J.B., Monteiro M.G. (2001). AUDIT: The Alcohol Use Disorders Identification Test: Guidelines for Use in Primary Health Care.

[63] Antich S., Rodilla V., Camañas L., Villagrasa V., Sanahuja M.A., Moreno L. (2006). Estudio descriptivo del consumo de psicofármacos en jóvenes: Necesidad de la Atención Farmacéutica en esta población. Pharm. Care Esp..

[64] Hudson W.W., McIntosh S.R. (1981). The assessment of Spouse Abuse: Two quantifiable dimensions. J. Marriage Fam..

[65] Foa E.B., Ehlers A., Clark D.M., Tolin D.F., Orsillo S.M. (1999). The posttraumatic cognitions inventory (PTCI): Development and validation. Psychol. Assess..

[66] Miller A.K., Markman K.D., Handley I.M. (2007). Self-blame among sexual assault victims prospectively predicts revictimization: A perceived sociolegal context model of risk. Basic Appl. Soc. Psychol..

[67] Expósito F., del Herrera M.C. (2009). Social perception of violence against women: Individual and psychosocial characteristics of victims and abusers. Eur. J. Psychol. Appl. Leg. Context.

[68] Pavlou M., Knowles A. (2001). Domestic violence: Attributions, recommended punishments and reporting behaviour related to provocation by the victim. Psychiatry Psychol. Law.

[69] Witte T.H., Schroeder D.A., Lohr J.M. (2006). Blame for Intimate Partner Violence: An attributional analysis. J. Soc. Clin. Psychol..

[70] Lerner M.J. (1980). The Belief in a Just World. The Belief in a Just World: A Fundamental Delusion.

[71] Schuller R.A., Smith V.L., Olson J.M. (1994). Jurors’ decisions in trials of battered women who kill: The role of prior beliefs and expert testimony. J. Appl. Soc. Psychol..

[72] Valor-Segura I., Expósito F., Moya M. (2011). Victim blaming and exoneration of the perpetrator in domestic violence: The role of beliefs in a Just World and ambivalent sexism. Span. J. Psychol..

[73] Horsfall J., Cleary M., Hunt G.E. (2010). Stigma in mental health: Clients and professionals. Issues Ment. Health Nurs..

[74] Little B., Terrance C. (2010). Perceptions of domestic violence in lesbian relationships: Stereotypes and gender role expectations. J. Homosex..

[75] Panchanadeswaran S., El-Bassel N., Gilbert L., Wu E., Chang M. (2008). An examination of the perceived social support levels of women in methadone maintenance treatment programs who experience various forms of Intimate Partner Violence. Women’s Health Issues.

[76] Llopis J.J., Castillo A., Rebollida M., Stocco P. (2006). Drug use and gender violence on addicted women in Europe. Keys for its comprehension and intervention/Uso de drogas y violencia de género en mujeres adictas en Europa. Claves para su comprensión e intervención. Health Addict. Salud Drog..

[77] Devries K.M., Child J.C., Bacchus L.J., Mak J., Falder G., Graham K., Watts C., Heise L. (2014). Intimate partner violence victimization and alcohol consumption in women: A systematic review and meta-analysis. Addiction.

[78] Logan T., Walker R., Cole J., Leukefeld C. (2002). Victimization and substance abuse among women: Contributing factors, interventions, and implications. Rev. Gen. Psychol..

[79] Alexander P.C., Tracy A., Radek M., Koverola C. (2009). Predicting stages of change in battered women. J. Interpers. Violence.

[80] Ferraro K.J. (1983). Rationalizing violence: How battered women stay. Victimology.

[81] Rolfe A., Orford J., Dalton S. (2009). Women, alcohol and femininity: A discourse analysis of women heavy drinkers’ accounts. J. Health Psychol..

[82] Califano J.A. (2006). Women under the Influence.

[83] Cascardi M., O’Leary K.D. (1992). Depressive symptomatology, self-esteem, and self-blame in battered women. J. Fam. Violence.

[84] Clements C.M., Sawhney D.K. (2000). Coping with Domestic Violence: Control Attributions, Dysphoria, and Hopelessness. J. Trauma Stress.

[85] Grubb A., Turner E. (2012). Attribution of blame in rape cases: A review of the impact of rape myth acceptance, gender role conformity and substance use on victim blaming. Aggress. Violent Behav..

[86] Miller B.A., Downs W.R., Galanter M., Begleiter H., Deitrich R., Gallant D., Goodwin D., Gottheil E., Paredes A., Rothschild M., Van Thiel D., Edwards H. (1995). Violent Victimization among Women with Alcohol Problems. Recent Developments in Alcoholism: Alcoholism and Women.

[87] Usher K., Bhullar N., Durkin J., Gyamfi N., Jackson D. (2020). Family violence and COVID-19: Increased vulnerability and reduced options for support. Int. J. Ment. Health Nurs..

[88] World Health Organization (2020). Mental Health and COVID-19.

[89] Volkow N.D. (2020). Collision of the COVID-19 and Addiction Epidemics. Ann. Intern. Med..

